# The development and feasibility of a group-based household-level intervention to improve preconception nutrition in Nawalparasi district of Nepal

**DOI:** 10.1186/s12889-022-12980-w

**Published:** 2022-04-06

**Authors:** Nadia Diamond-Smith, Ashley Mitchell, Alia Cornell, Minakshi Dahal, Lakshmi Gopalakrishnan, Mallory Johnson, Sheri Weiser, Mahesh Puri

**Affiliations:** 1 Department of Epidemiology and Biostatistics and Institute for Global Health Sciences, University of California, San Francisco, 550 16th Street, 3rd Floor, San Francisco, CA 94158 USA; 2 University of California, San Francisco, 550 16th Street, 3rd Floor, San Francisco, CA 94158 USA; 3 University of California, Los Angeles, USA; 4 Center for Research on Environment Health and Population Activities (CREHPA), P.O.Box. 9626, Kusunti (near Yatayat office), Lalitpur, Nepal; 5 School of Public Health, University of California, 2121 Berkeley Way, Berkeley, USA

**Keywords:** Intervention, Nutrition, Pregnancy, Preconception, Households, Norms

## Abstract

**Background:**

In a setting such as Nepal with malnutrition and persistent poor maternal and infant health outcomes, developing interventions to improve the nutrition of preconception and pregnant women is essential.

**Objective:**

The objectives of this paper are to describe the full design process of an intervention for newly married women, their husbands, and mothers-in-law to improve maternal nutrition and gender norms, and findings from the feasibility and acceptability pilot.

**Methods:**

In this paper we describe the three phases of the design of an intervention in rural Nepal. We first conducted a mixed methods formative phase which included in depth interviews with newly married women, their husbands and mothers-in-law (*N*=60) and a longitudinal study for 18 months with 200 newly married women. We then designed of a household level, group, intervention, in close partnership with community members. Finally, we conducted a pilot intervention with 90 participants and collected both pre/post survey data and in-depth qualitative interviews with a subset (*N*= 30). All participants from all phases of the study lived in Nawalparasi district of Nepal. Qualitative data was analyzed using a thematic analysis, with inductive and deductive themes and quantitative data was analyzed using descriptive statistics.

**Results:**

Our formative work highlighted lack of awareness about nutrition, and how women eating last, limited mobility, household and community inequitable gender norms and poor household-level communication contributed to low quality diets. Thus we designed Sumadhur, an intervention that brought groups of households (newly married wife, husband, and mother-in-law) together weekly for four months to strengthen relationships and gain knowledge through interactive content. We found Sumadhur to be highly feasible and acceptable by all respondents, with most (83%) attending 80% of sessions or more and 99% reporting that they would like it to continue. Pre/post surveys showed a decrease in the proportion of women eating last and increase in knowledge about nutrition in preconception and pregnancy. Qualitative interviews suggested that respondents felt it made large impacts on their lives, in terms of strengthening relationships and trust, understanding each other, and changing behaviors.

**Conclusions:**

We show how a designing an intervention in close partnership with the target recipients and local stakeholders can lead to an intervention that is able to target complicated and culturally held practices and beliefs, positively benefit health and wellbeing, and that is very well received.

**Trial registration:**

ClinicalTrials.gov NCT04383847, registered 05/12/2020

**Supplementary Information:**

The online version contains supplementary material available at 10.1186/s12889-022-12980-w.

## Background

Gender inequality and low levels of empowerment among women contribute to poor health outcomes for women and children in South Asia [1]. Indicators of women’s status and autonomy, including the gender development index, are lower in South Asia than other low and middle income countries (LMICs) and strongly correlated to child stunting and wasting, among other adverse outcomes [1]. Low maternal education, limited ownership of land by women, poor female labor force participation, and low decision-making power among women are further associated with adverse child health outcomes and poor care-seeking in Nepal [2]. A preference for sons means that daughters born into families with more siblings are at increased risk of acute and chronic malnutrition and poor growth [3]. Restrictive gender norms and practices are associated with poor maternal and child health outcomes through delayed/low care-seeking, poor nutrition, depression, etc. Exposure to physical violence and abuse (such as food restrictions and denial of health care) from husbands and in-laws perpetuates inequality and worsens maternal, infant and child mortality and morbidity [4–6].

Young, newly married women in the preconception stage are at the lowest status in their marital households. In much of Nepal, marriages are arranged or at least heavily involve the family. Newly married women often eat last, cook and serve all other household members, and have limited mobility or decision-making power [7–9]. Married women who have not yet given birth or have not yet had a son have the lowest status in their marital households and are often confined to their homes, restricting their ability to seek health care. They are further harmed by customs such as not speaking in front of household elders and being at the will of their mothers-in-law [10, 11]. About half of women in Nepal experience a pregnancy within the first year of marriage, making nutrition in early marriage critical [12]. Newly married women in food insecure households often have less access to diverse foods in their husband’s, compared to their parents’, home [10]. However, better relationships between daughters-in law and mothers-in-laws have been found to mitigate the association between household food insecurity and lower consumption of high-quality foods upon moving into the marital home [10].

Most interventions aiming to improve pregnancy and birth outcomes globally, including in Nepal, focus on women once they are pregnant or before marriage (in adolescents), thereby neglecting newly married women in the important preconception period. Group-based intervention models have been shown to be successful at increasing reproductive and other health knowledge, as well as empowerment, for women in South Asia [13–17]. Interventions promoting gender equality and challenging restrictive gender norms can positively influence women’s health [18]. Group interventions are especially important, and impactful for addressing household and community gender norms, and have been found to increase health knowledge in South Asia [13–15, 19]. There have been a large number of group pregnancy interventions, and many have successfully improved iron folic acid uptake, consumption of nutritious foods, and reduced anemia [17].

Engaging family members is also important in South Asia, where co-residence early in marriage is still common and decisions are often made at a household level [20]. A 2020 systematic review of nearly 60 programs targeting gendered social norms and health found that more than half specifically engaged males to decrease violence and redefine household roles and responsibilities, though the majority (67%) named females as the intended primary beneficiaries. Approximately three-quarters (74%) of evaluations identified significant changes related to both health and gender [18]. Gaps remain, as only few evaluations measured nutritional outcomes [18]. Household interventions in Nepal involving women, their husbands, and in-laws have demonstrated improved livelihoods for young married women, increased earnings and savings, and reduced food insecurity [16, 21]. However, we found no studies that targeted whole households; instead, studies to date recruited women, mothers-in-law, and husbands from separate homes, or only engaged a subset of family members.

To address the intersecting community, household, and individual factors contributing to poor nutrition and early pregnancy among newly married women in Nepal, we conducted formative research, followed by a community-based participatory design phase, to develop a group-based household level intervention, named *Sumadhur* (meaning “Best Relationship”). *Sumadhur* is a group intervention that engages members of households with a newly married woman, her husband and her mother-in-law weekly over four-months to provide interactive, informational content about nutrition and women’s health, address inequitable gender norms and practices, strengthen household relationships and communication, and improve the status of newly married women.

## Methods

All parts of this study were conducted in Nawalparasi district of Nepal, near the Indian border. This district was selected because it has relatively low indicators of women’s status and empowerment, described in more detail in previous publications from this study  [22].

### Part 1: Preliminary research

Our team conducted mixed methods, formative research in the communities in which we would develop our intervention. We first conducted in-depth qualitative interviews with 20 household triads (newly married women, their husbands, and mothers-in-law). We then conducted an 18-month longitudinal study with 200 newly married women, followed every six months (four rounds of data collection) between 2018 and 2020. Only two women that were approached for the longitudinal study refused to participate and one household that was approached for the qualitative study refused to participate. More details about study design, recruitment, and data collection for this formative work, along with other findings not presented here, have been published elsewhere [10, 22–24]. For both parts of this research participants provided written consent, including consent for the in-depth interviews to be audio recorded.

#### Analysis

Descriptive statistics from the baseline survey data are presented (frequencies, percent). Details of the analysis of the qualitative data has been published elsewhere [22, 23]. In summary, data was coded by a team of researchers from both Nepal and the US, who were in constant communication to consider and address any potential bias. A subset of interviews were also double or triple coded. Please see supplementary files for the interview guide, codebook and themes. For the purpose of this paper, we reviewed the codes and themes, both looking at the data as individuals and then as triads.

### Part 2: Development of *Sumadhur*

Through a community engaged design approach, we collaboratively developed and piloted *Sumadhur,* an intervention to influence these pathways among newly married households in Nawalparasi District of Nepal. First, throughout the course of the mixed methods formative study described above (more than a two-year period), we held bi-annual meetings with a District Advisory Committee (DAC), initially comprising five members and later extended to 11 members to accommodate more community stakeholders. 1 Their main roles were to suggest study areas for the study and intervention, participate in dissemination meetings, suggest potential interventions based on findings, support the core team members to implement the study and monitor the intervention activities.

Based on the analysis of data from the formative phase and input from our district advisory committee, we began drafting a list of educational sessions. At this point, we also reached out to other researchers and implementers working in Nepal and India who had group interventions that addressed some of the topics that we were covering. Many of these groups shared their intervention protocols with us and gave us permission to adapt some of their content/materials to our population and target.^2^ Our local implementing partner, Vijay Development Resource Center (VDRC) and research partner, Center for Research on Environment Health and Population Activities (CREHPA) held a series of meetings to decide on the final contents of the intervention. We had three day-long intensive meetings during the development and finalization of intervention content phase and a five- day intensive training for group facilitators before the implementation. Through this process we developed our intervention, *Sumadhur.*

### Part 3: Feasibility and Acceptability Pilot

*Sumadhur* was piloted in six villages among six groups, with five households per group (for a total of 15 people per group and 90 people in the intervention: 30 newly married women, 30 husbands and 30 mothers-in-law). This sample size was determined to give adequate number of responses per participate type (30 each) and feasibility at the time of roll-out given the COVID-19 pandemic. We conducted pre-post surveys with all 90 participants and in-depth qualitative interviews with a subset of 30 participants (all three members from 10 households, at which point the team felt that saturation had been reached).

Group sessions were organized in a convenient location to all the participants such as ward offices, schools, local government building or office hall (within their village, not more than half an hour walking distance, within approximately three-kilometer radius). VDRC hired three facilitators with Bachelor’s degree level of education to be trained for the intervention. Two facilitators, one each for Sunwal Municipality and Palhinandan Rural Municipality, were selected and trained. The third facilitator was responsible to coordinate and help facilitators in both the municipal areas. The facilitators were provided a five-day intensive training before the launch of the intervention. The training was facilitated by the core team members from CREHPA and VDRC. During the training, the facilitators were oriented on facilitation skills, content, and mock sessions. A field officer from CREHPA attended most of the sessions. Core team members from CREHPA and VDRC also observed selected sessions and discussed lessons learned and provided feedback to the facilitators to improve in the subsequent sessions.

#### Analysis

For the quantitative data, we use descriptive statistics (frequencies and percent). Primary feasibility and acceptability measures include satisfaction (five-point Likert scale), if a participant would recommend to a friend, number of sessions attended, and main barriers. For the qualitative data, we used thematic analysis because this is a useful approach for understanding what themes respondents bring up frequently or in depth, and understand the interrelationship between themes across participants [32]. Using inductive and deductive approaches [33], themes that pertained to the concepts of interest were identified and additional concepts or themes that emerged spontaneously during the interviews were also included. Once the key concepts were identified, a codebook was developed by a three-person team (NDS, AC, AM) through an iterative process. A quarter of interviews were double coded, and where discrepancies arose, these were discussed until agreement was reached.

All phases of this study received ethical approval from the University for California, San Francisco and the Nepal Health Research Council.

## Results

### Part 1: Formative research

Women were marginalized in terms of eating practices and diet, with almost half (47.5%) reporting that they ate last always or most of the time and 88.5% not meeting minimum dietary diversity standards (Table 1).

**Table 1:** Formative quantitative findings from formative phase survey just after marriage

	*N*=200 (%)
Eats last usually or all of the time	95 (47.5)
Did not meet minimum dietary diversity (5 or more food groups)	177 (88.5%)
Has been to the market since marriage	72 (36.0%)
Discussed how many children desired with husband	85 (42.5)
Feel comfortable talking to husband about sex	64 (32.0)

In the qualitative data, women described not eating enough and not eating foods they preferred, with one wife explaining “*No one cares if I eat, what I eat. No one asks how I am feeling…. I feel no one cares about me.” (Formative Phase Wife #1, Age 18).* All household respondents described ordered eating patterns, with newly married women eating last. This was often explained by “culture” or “tradition” or as a practical solution to the need for someone to be serving the food, combined with a willingness on the part of the wife to eat less, as described by one husband:

*I: So, all four of you eat together?*

*R: No, my wife doesn’t eat with us. She eats after we all finish eating.*

*I: Why is that so?*

*R: This is because we may need to add something when we eat. If she is eating as well, it becomes difficult for her to give us additional food. Therefore, we eat at first and after we finish eating, she eats it.*

*I: So, does the food becomes sufficient for her?*

*R: Yes, it does. We usually tell her to cook food if it is insufficient. But she eats less and tells the food is sufficient for her. (Formative Phase Husband #8, age 20)*

Some household triads reported positive and loving relationships, but many wives felt that they were not cared for “*He doesn’t help me in my work since he has married me and brought me here…My husband never asks me anything regarding my health and wellbeing. (Formative Phase Wife #11, Age 20)”*

Quantitative data supported low levels of communication between spouses, with less than half of wives reporting having discussed the number of children they wanted with their husbands (42.5%) and a third (32%) feeling comfortable talking to their husbands about sex (Table 1).

Mobility was also strictly limited, with only 50% having ever left the house since marriage. In depth qualitative interviews highlighted how isolated and lonely wives were, as described below:

*R: I like to go but who allows me to go? I don’t have anyone who accompanies me. My husband does not have much free time, nor does he have any interest. I am helpless. I also don’t have free time due to household work. If I get some free time, then I sleep. How can I have free time after marriage? I mean our life becomes imprisoned. We have to live like a prisoner. It is really hard to go for shopping after marriage.*

*I: Like a prisoner?*

*R: Yes, I mean like a chained animal. When you can’t do whatever your heart wishes, when you can’t go wherever you want to go.*

*I: Why?*

*R: In our society, people do not like when a daughter-in-law goes outside her house. It is not considered as a good thing. All we have to do is sit behind the curtain, do household chores. I have started to talk nowadays. After marriage, our life is not as before.*

*I: How do you feel seeing this?*

*R: After seeing this, I really feel sad about it. Before when I was in my maternal house, I used to go outside, eat and have fun. But now I feel that my life is limited within one house. I mean sometimes I really want to cry thinking about it (saying emotionally) (Formative Phase Wife #2, age 18)*

This restriction in mobility led to barriers to women getting information about nutrition:

*I don't have enough information about food. I don't know what food should we take at what time. To have this information I should go out of the house, but I am not allowed to go anywhere out of the house. My husband works abroad. There is not much work in the house, there is only one person who earns money and he needs to look after seven members. Our earning are not sufficient, so it is difficult to fulfill our needs. No one else in the family is employed. We don't have good education on what we should eat, we only know that we should eat rice, lentils, but we don't know about other foods. (Formative Phase Wife #13, age 18)*

Restricted mobility was also seen as a potentially large barrier to women being able to participate in an intervention, especially alone:

*I: Do you think your wife will be willing to participate in the program?*

*R: Yes, she will be interested to participate if the program is within the house. If it is outside, she will not be able to participate.*

*I: Why can't she participate?*

*R: It is not in our culture to allow newly married women to go outside the house.*

*I: And will your mother participate in such programs?*

*R: Yes, she will be willing to participate.*

*I: Will she be allowed to go outside the house and participate?*

*R: Yes, she will be. She doesn't have to ask anyone. She can simply inform us and go in the program.*

*I: So when can your wife go outside the house and participate in the program?*

*R: It depends; the culture here is that we have to stop her from going outside as long as we can. The people here aren’t educated and they think that women should be kept at home and men should go outside and be the bread earner. (Formative Phase Husband #9, age 21)*

In terms of the design of the intervention, respondents reported that they would prefer an intervention that engaged household members in addition to the women, and that it not be moderated by a community health care worker. Furthermore, involving husbands and mothers-in-law was also seen as an acceptable way for wives to be allowed to leave the home.

### Part 2: Development of Sumadhur

Our findings highlighted nutrition as a high need area, which also aligned with our goals of improving preconception health and wellbeing of women (and ultimately maternal and infant health). These findings, in combination, suggested that strengthening household relationships by bringing the three key players together (newly married women, husbands and mothers-in-law) was important and could be a vehicle to address household and individual practices. Bringing groups of households together could further get at community norms around expectations of women’s role and eating dynamics. We hypothesized that strengthening relationships and addressing inequitable norms could improve women’s household status, increase mobility, and increase access to food.

Based on the preliminary findings, and through the community-engaged process described above, we developed *Sumadhur,* a four-month long, weekly group intervention for triads (wives, husbands, and mothers-in-law) that covers nutrition, anemia, intrahousehold food allocation, prenatal health and pregnancy care, gender inequitable norms and practices, fertility planning and contraception, and couples and household relationship dynamics (Table 2). Each session combined educational information with interactive topic-related games and activities that helped build relationships and break social and gender norms. As can be seen in Table 2, most sessions included all three household members, but a sub-set only included the wife and husband, for more sensitive topics. Detailed discussion and feedback with our partners and DAC informed the decision about which sessions should all have household members, the number of households that should be in each group, additional input about specific session content. Except in two sessions, all sessions were moderated by the trained moderators from VDRC; those two other sessions had health workers come in to provide more detailed information on family planning and biology/menstruation (Sessions 14 and 10).

**Table 2:** Overview of topics and activities in Sumadhur

	Title	Topics and Brief Summary of Activities
1	Introduction	-Program welcome; participant introductions; ground rules and expectations
2	Marital Relationships (*Couples Only*)	-**Topics:** Healthy Relationships; Defining the Ideal Partner -**Activities:** practice identifying relationships and behaviors as “healthy” “unhealthy” or “depends”; guided group dialogue about ideal qualities of partners, gender roles, and relationship equality
3	Household Relationships	-**Topics:** Newly Married Life; Showing You Care Loudly -**Activities:** comparison of newlywed experiences, comfort, tasks, and food security; practiced identifying example scenarios of caring
4	Gender Inequity	-**Topics:** Gender Roles; Men and Boys as Drivers of Norm Change; Daily Routines -**Activities:** sharing of gender-specific oppressions and inequalities; guided group reflection to gender role scenarios; collaborative assessment of uneven burden of daily household chores
5	Gender Roles and Household Eating Patterns	-**Topics:** Gender Roles; Order of Household Eating -**Activities:** participant skit demonstrating the consequences of uneven responsibilities impacting meaningful food access; small group activity in which members distribute increasingly limited foods and discuss who is prioritized
6	Nutrition in Pregnancy	-**Topics:** Importance of Nutrition During Pregnancy; Dietary Diversity During Pregnancy -**Activities:** comparison and discussion of photos of depicting intergenerational outcomes of nourishment; guided group reflections about images conveying food and diet variety
7	Problems and Barriers to Nutrition in Pregnancy	-**Topics:** Nutrition During Early Pregnancy; Barriers to Adequate Nutrition During Pregnancy -**Activities:** dialogue responding to images of common antenatal barriers to nutrition (i.e. nausea, heartburn, etc.); small group discussions identifying barriers and solutions to eating diverse foods, iron and folic acid supplementation, and deworming
8	Preconception Period	-**Topics:** Importance of Nutrition in the Preconception Period; Reproductive Goals -**Activities:** dialogue following a metaphor comparing the cultivation needed for wheat and for preconception/pregnant women; individual plotting and then comparing of reproductive life plans
9	Norms around fertility and birth spacing	-**Topics:** Fertility Norms; Birth Spacing; Son Preference -**Activities:** discussion about community childbearing norms; storytelling to convey benefits of birth spacing; discussion of statements about social/ cultural significance of the sex of a child
10	Biology and Menstruation	-**Topics:** Introduction to Menstruation; Menstrual Hygiene -**Activities:** gender-specific discussions of menstruation and facilitator review of the biology of fertilization and sex determination; guided dialogue about menstrual hygiene habits
11	Pregnancy Care and Safe Delivery	-**Topics:** Importance of Antenatal Check-ups; Illness and Danger Signs During Pregnancy -**Activities:** interactive review of the purpose and frequency of antenatal check-ups; guided dialogue about danger signs necessitating a hospital visit and development of household-specific birth plans for safe delivery
12	Anemia and Iron and Folic Acid	-**Topics:** Birth Spacing; Anemia and Iron and Folic Acid -**Activities:** engagement in a metaphor of strength and weakness followed by a discussion about the intergenerational, gendered effects of anemia and possible solutions; guided dialogue and debunking of myths related to iron folic acid
13	Stress and Anxiety	-**Topics:** Adverse Events and Stress -**Activities:** small group discussions about causes and consequences of adverse events (i.e. unemployment, illness, relational conflict); guided practice of breathing exercises and discussion of their benefit to reduce stress and anxiety
14	Family Planning, Miscarriage, and Abortion (*Couples Only*)	-**Topics:** Method of Family Planning; Misconceptions Regarding Contraceptives; Miscarriage and Abortion -**Activities:** interactive lesson from health worker about all methods of contraception; discussion of myths and couple-specific conversations; guided dialogue on safe abortion services in Nepal
15	Intimate Partner Violence (*Couples Only*)	-**Topics:** Introduction to Intimate Partner Violence; Effects of Intimate Partner Violence on Women and Her Children; Ways to Prevent and Control Intimate Partner Violence -**Activities:** sorting of scenarios depicting intimate partner violence vs. healthy relationships and an interactive lesson defining/ describing intimate partner violence; guided dialogue about abuse and outcomes through the antenatal period; discussion of prevention and relevant laws/ policies
16	Closing: Making Sun	-**Topics:** Recalling the Sessions; Experience Sharing and Reflection -**Activities:** reflective sharing of key program take-aways; invitation to share experiences and partake in a celebratory meal

### Part 3: Intervention pilot results

i. Feasibility and acceptability:

A total of 44 households were approached initially. Ten households did not agree to be a part of the study at the first approach citing various reasons (inability to manage time, no good relation among wives and mother-in-law, the wife having gone to her maternal place for a long time). Four households backed out at the last moment citing fear of COVID-19. Therefore, an additional four households were approached. A total of 30 households consented to take part in the intervention. One household was again replaced after presurvey as the husband obtained visa for foreign employment. Those households were divided into six groups (each group comprising of five households). The sessions were conducted weekly in five groups, however, one group requested that they be conducted biweekly. Most of the sessions were conducted in the daytime as per the participant's choice and convenience. The sessions lasted an average of 81 minutes (73-91 minutes).

A total of 31 households participated, however, only 28 husbands answered the surveys. One of the husbands had gone abroad immediately after the pre-survey and did not attend any sessions. Another husband attended two sessions and then went abroad. Table 3 shows the socio-demographics of the participants.

**Table 3:** Feasibility and acceptability

	**Wives (*n*= 31)**	**Husbands (*n*=28)**	**MIL (*n*=31)**
**Number session attended (max 16)**	14.35 (SD 3.5)	15.1 (SD 2.5)	11.9 (SD 2.5)
**Would recommend**			
**Yes**	30 (97%)	28 (100%)	30 (97%)
**No**	0	0	0
No answer	1 (3%)	0	1 (3%)
**Satisfaction**			
Very satisfied	27 (90%)	28 (100%)	21 (70%)
Somewhat satisfied	3 (10% )	0	9 (30%)
Somewhat	0	0	0
Unsatisfied	0	0	0
Very in satisfied	0	0	0
No answer	1	0	1

*Sumadhur* was acceptable and feasible, with 97-100% of participants reporting that they would recommend it to a close friend and that they talked to someone about something they learned (Table 3). Most (83%) attended 80% of sessions or more, and the majority (73%) of participants reported “no difficulties” in attending sessions; participants identified health issues (*n*=12), personal work obligations (*n*=4), and personal household obligations (*n*=4) as the primary attendance barriers. Satisfaction rates were high, with 100% or respondents being very or somewhat satisfied. Additionally, there was a desire for the intervention to continue, with 99% reporting that they would like something like this to continue in the future. Additionally, despite initial hesitancy about combining husbands, mothers-in-law, and wives, 95% of wives reported that it felt “good” to attend session with their in-law.

Respondents also enjoyed the program, including interacting with others in their community, as one wife explained how it built relationships with others in her community:

*I felt very good to participate with other community members. It felt like being in a family when people from different community, caste and family structure participated in the training. I didn’t feel that we represented different family or community. It felt as though we all were from the same family and were attending the training for mutual benefit. Maybe I felt that because everyone was cooperative, understanding and helpful….They all had their own definition and understanding on the topics covered. …in earlier days, my neighbors didn’t call me by my name. But after attending the training, they call me and ask me about the things I've learned from the training. I used to tell them the things I've learned. I also told them to participate in such trainings in future. After the training, I felt that the community here is very supportive. Other women of my age come to me and ask me about the training. I tell them about the things I've learned and also suggest them to maintain peace and mutual cooperation in the family. (Pilot Wife 13, age 21)*

A husband also discussed how he appreciated the group dynamics and engagement of other community members:

*R:I felt comfortable to be in a group. There were other members with whom we could interact and know them better. Had it been only my family in the training, it would have been less interactive. In the institute, we try to have more students to make the class better. Similar approach was used in the training and I liked that part. It wasn’t uncomfortable with other community members. Also, it is very important to give training to community members as well. Here, the community follows traditional practices. I think such training programs will help to change their thought process.*

*I: What do they follow?*

*R: In our community, daughters-in-law cannot come outside freely, woman cannot go outside their house to work, mothers-in-law and daughters-in-law don't interact much etc. Such things need to be changed. It was somewhat similar in my household as well. But after the training it has changed a little. As I said earlier, we eat together and interact much more than before. My wife and my mother interact more and this makes me feel good. (Pilot Husband #25, age 19)*

Despite initial concerns about constraints on women attending due to restricted mobility, *Sumadhur* provided an opportunity for some to leave their homes for the first time since marriage:

*R: I was shy to attend the program at the beginning, which later on decreased. I couldn’t understand few words spoken by the facilitator, but I hesitated to ask for clarification as there were other male participants in the program. With time, I felt comfortable and my hesitation also decreased.*

*I: Do you want to say anything else?*

*R: I had not stepped outside of my home post marriage. I felt very happy to step outside of my home to attend this program. You learn some new things when you step outside of home. Attending program has increased my knowledge, I came to know about many things. I have also developed confidence for speaking. This kind of program raises awareness among people. I am very happy to be a part of this program. (Pilot Wife #3, age 20)*

In addition to immediate dietary changes, participants liked the information related to nutrition. When participants were asked which topics they found most useful, anemia and iron folic acid were reported most often by 61% (*n*=55) of participants. Topics including household relationships, gender inequality, household eating patterns, and stress and anxiety were also among the top five most useful.

We found that while engaging with sensitive and stigmatized topics together was new for many households and groups, by the sixth session comfort and engagement was reported by the facilitators to have increased across the participants.

*Even though people are educated, they feel shy in front of their family members. After participating in the training, the participant gained confidence to speak and express what they feel to their family members. The training helped in their personal development. One participant did express her feeling to me after the training. She said that she used to feel shy in front of her family members. After attending the session, her mother-in-law includes her in family conversation and encourages her to speak. In my opinion, through the training we brought closeness among daughters-in-law and mothers-in-law. If they can continue this bonding, this will help the family to become strong. (Facilitator)*

ii. Preliminary impact of the intervention

**Fig. 1:**
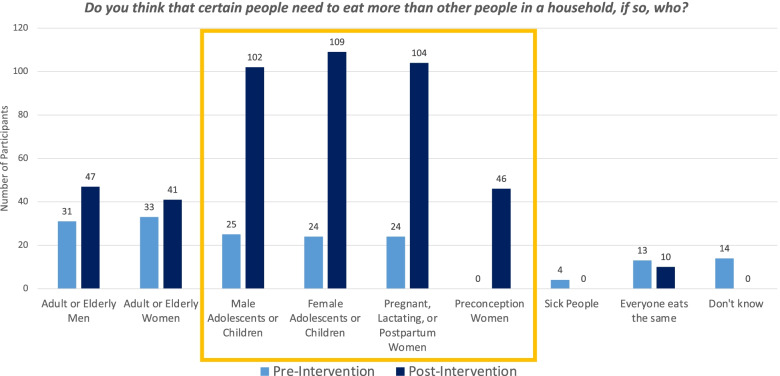
Proportion of respondents agreeing that certain people need to eat more than others, pre and post intervention

As a result of the intervention, nutritional knowledge and practices improved. There was increased awareness of the need for preconception, pregnant, lactating or postpartum women and adolescents to eat more (Fig. 1). Eating patterns also shifted, with a decline in the number of daughters-in-law reporting that they ate last (from 43% to 3%) and increase in the proportion reporting that the household ate together usually or all of the time (from 37 to 52%) (Table 4).

**Table 4:** Change in eating practices pre and post intervention

	Before Intervention *n* = 90	After Intervention *n* = 90	*p*-value
Does everyone in the household eat at the same time together?			
Never	41% (*n*=37)	10% (*n*=9)	<0.001
Rarely or Sometimes	22% (*n*=20)	38% (*n*= 34)	
Usually or All the time	37% (*n*=33)	52% (*n*=47)	
Newly married woman ate last always or most of the time (newly married women only)	43%	3%	<0.001

Overwhelmingly, respondents had absorbed information about what types of foods women should eat before and during pregnancy. Intertwined with increased knowledge about nutrition itself, respondents described changing dynamics around support for women, order of household eating and awareness of the importance of strengthening relationships, as described in the two quotes below:

*Nowadays, my mother-in-law helps me a lot with my household work. As I am pregnant now, she asks me to eat egg daily saying it’s good for my health…Even if I don’t say anything, she tells me to have snacks saying that I might be hungry. She also says that I should eat more in this condition. She says eating frequent and nutritious food will be beneficial to both me and my baby inside me. (Pilot Wife #16, age 23)*

*My wife is pregnant, she also learned things related to pregnancy and childbirth.... Now, both my mother and my wife are trying to establish a good relationship. She is eating nutritious food and also asks for my help. In earlier days, she didn’t ask for my help in any work. I also have been helping her in household work. ...we all eat together now...My wife eats more nutritious food like green vegetables, egg, meat, cereals and lentil. We tell her to rest more and prevent her from doing any heavy work. If there’s any important work, we take suggestion from other family members before doing it (Pilot Husband #30, age 27)*

Myths that mothers who eat too much or take iron tablets will gain weight, and other misconceptions about nutrition and health, were also dispelled, as this respondent explained:

*I used to hear that to prevent unborn baby from gaining weight, pregnant mothers were prevented from eating nutritious food and iron tablets. But I came to know that all these weren’t true. We have to eat nutritious food, take adequate rest and consume iron tablets on time. I’ve been implementing these things in my life as well. (Pilot Wife #13, age 21)*

Respondents also got to know each other better, for example one husband described learning something surprising about his wife: *“I didn’t know that my wife was somewhat scared of childbirth. She said that she is scared of complications during her delivery. We told her not to stay worried as staying worried might cause health problems during pregnancy.” (Pilot Husband #30, age 27)*

Two mothers-in-law described how it helped build a relationship with their new daughters-in-law, and how the family-group approach made them less shy about participating:

*Going together with my son and daughter-in-law, I came to know many things. It was easier for me to go together with my son and daughter-in-law. I would have been shy to talk too if I had gone alone, but going together with them became easier for me…..I have felt changes even if it’s a little. There are changes in everything regarding behavior of the family members, working environment, eating habit, conflicts. I had always wondered how would my daughter-in-law be. As she was recently married, I had not known her well. I got an opportunity to know her while going to the program. We used to go together, talk on the way and discuss about the things discussed after we would come back. We perform household work together, talk to each other and share our things. I had a negative attitude towards daughter-in-law earlier which has changed now. My daughter-in-law treats me well and so do I. (Pilot MIL #9, age 54)*

*I have found it so good that I cannot express in words (*She said with much of excitement, and with happiness*). I really liked the part where we learnt that we should not discriminate between a family who has sons with those who have daughters only. But, in villages if the family has female child only, people talk a lot behind their backs. It is not just sons who can take care of the parents even daughters can look after them….. When I went along with my son and daughter-in-law, if there were things that I didn’t understand, my daughter in law would make me sit and used to explain by saying that we were taught these things today. And when we went together, the villagers would also say that it is so nice seeing her going with her son and daughter in law to the program. I used to feel very good. (Pilot MIL #25, age 43)*

A newly married women shared about how her relationship with both her husband and mother-in-law changed:

*R: Yes, there has been changes. The behavior towards me when I was newly married has changed after taking part in the intervention. I had to perform all the household chores by myself before but the scenario has changed now. Everyone helps with household chores. There is equal distribution of food among all the family members. We don’t fight with each other. There has been changes in husband’s and mother-in-law’s behavior. Previously, my husband used to force me for sexual relation but he doesn’t do it now. He asks about my health, respects me and loves me. I am happy now.* *I: Are you surprised listening to views of your family members?*

*R: I am surprised seeing the love and care towards me nowadays. I used to feel being neglected previously but now husband, mother-in-law, sister-in-law, brother-in-law care and love me. They help me in household chores, everyone eats together. I have understood that If a person gets good information then it is beneficial to whole family. (Pilot Wife #3, age 20)*

All participants felt that others in their communities and other districts should have access to this program. Participants also raised that other family members should be invited to join, such as other daughters-in-law in co-resident households with multiple sons and families living together, or, most commonly, the father-in-law:

*People in the villages are still a bit ignorant, and my father-in-law comes home drunk sometimes. Well, my husband, my mother-in-law are attending the program, but my father-in-law does not understand, he needs to be taught, even he needs to be invited to the program. Everyone in the home should participate in such programs, and they should be informed about everything, such as “what would make it good and better at home”. Well, it so happens that at my home, we often have arguments and I want to make them understand (Pilot Wife #26, age 20)*

*My father-in-law was very curious to know about this training. He used to ask us every time. If he attends the training, he can learn many things from it. This is because, the older generation mindset takes time to change. Family and society cannot change and become progressive until and unless such older generation's mindset changes. It is important to bring changes in belief system of the head of the family. In order to bring such change, training like "Sumadhur" can play an important role. If head of the family bring change in their belief system, other members also follow it. Therefore, I think it is very essential to include father-in-law in the training. (Pilot Wife #13, age 21)*

## Discussion

Targeting triads from multiple households in an intervention designed to improve preconception and pregnancy nutrition as well as strengthen relationships and address inequitable gender norms was found to be feasible, acceptable and showed improvement in behaviors. Participants were overwhelmingly positive, finding that this intervention made marked changes on their lives. Furthermore, even culturally entrenched behaviors such as the order of eating in the household [9], were seen to be malleable through this four-month intervention, suggesting that perhaps some practices that have hitherto been assumed to be hard to change might actually be able to be shifted when people have additional information and understand each other’s perspectives.

Despite restricted mobility of newly married women in this setting, and the potential for this to be a barrier to an intervention that occurred outside of the home and with other community members, we had high rates of participation from newly married women. Not only this, but the opportunity to participate in this intervention served as a way for newly married women to be able to leave the house, prompting downstream effects on their autonomy. A related unexpected finding was the impact of the intervention on mothers-in-law. While we hoped that the intervention would help build relationships between mothers-in-law and their daughters-in-law, as it did, we also found that mothers-in-law felt empowered by the opportunity to participate in the intervention, leave the house, and interact with others in this new way. Few interventions engage, much less target, mothers-in-law (or older women), and this population deserves more focus and attention [34]. We identified one past intervention that engaged married women, their husbands and mothers-in-law to reduce violence, address gender norms, and provide economic opportunities for young women, which was found to be effective at impacting some of these outcomes [35]. Clearly, there is a need for more such inclusive approaches.

Overall, the intervention was successfully implemented and welcomed by district level stakeholders. Therefore, it could be scaled up with few minor modifications in the content, approach and preparation of information, education, communication/behavior change communication materials. The modality of conducting sessions weekly seemed appropriate as it does not overburden the participants with information. Since the sessions were conducted in the local language, development of materials in local language would have an additional advantage. It is possible that an intervention such as this may be more interesting and applicable to rural and relatively less educated participants, and thus, more research is needed about its applicability in a more urban setting.

Part of the success of this intervention was the community engaged process, from formative research, to design of the intervention, and roll-out. The local government and district level stakeholders showed their commitment to support this intervention, therefore the engagement of such stakeholders from the early stages of the design would be beneficial and key to successful implementation of future intervention such as this. Additionally, the design and content of the intervention was derived directly from the findings of the in-depth, multi-year formative work, which helped make the intervention salient to participants. Our team was able to shift focus based on the voices of community members, for example, our initial design idea had been an intervention in households given to household members individually by community health workers and our formative phase helped us see the need for different types of facilitators to provide information in a group structure.

The community based participatory design and evaluation of this intervention, with rigorous formative and pilot research, are strengths. However, as with all studies, there are several limitations. First, this study was conducted over a 4-and-a-half-year period, and thus, some of the findings from the early phase might not have been relevant 4 years later, as social and gender norms, barriers to food, etc. may change. The last phase of the study (pilot of the intervention) occurred during the COVID-19 pandemic, which may have heightened food insecurity, increased family stress, and had other impacts that might have influenced both the implementation and evaluation process. This study was conducted in one region of Nepal, with specific gender and social norms, and thus might not be generalizable to other parts of Nepal. However, part of our goal in describing our whole design process is to help future implementation designers and researchers to replicate this process as much as possible. Finally, the interpretation of the qualitative data and other decisions about the design of the intervention could have been biased by members of our teams’ cultural background or areas of interest—we strove to limit this by having continual engagement from our community stakeholders, a bi-national team, and strong team communication and reflection.

From a methodological perspective, the community engaged approach outlined in this paper could be used by those interested in designing interventions that are reflective of the needs of the community and that address complicated social norms and practices. NGOs, government organizations or policy makers could also adapt this content to similar topics or populations or neighboring regions—additional details about the intervention content, including the manual itself, are available upon request. Broadly, interventions such as this can have lasting improvements on people’s lives by not just increasing knowledge, but by fundamentally strengthening the relationships that determine behavior and quality of life.

## Conclusion

Through a community-engaged process informed by mixed methods research, we were able to design a feasible and acceptable intervention that not only addressed norms and practices, but actually through its very structure (bringing young newly married women out of the house and bringing households together) challenged these norms and led to empowerment, strengthened relationships, and changes in health knowledge and behavior.

## Supplementary Information

**Additional file 1.** Codebook and themes.

**Additional file 2.** Post-intervention interview with participants:semistructured qualitative interview guide.

## Data Availability

The datasets used and/or analysed during the current study are available from the corresponding author on reasonable request.

## Footnotes

1 The DAC was comprised of representatives from district public health office (head), maternal and child health focal person of district public health office, civil society organization working on nutrition, and women and child health, local level elected ward members, etc.

2 Specifically, we learned from the RANI project (India) [25], the MIRA project (mother and infant birth activities, Nepal) [26–28] , the CHARM project (India) [29, 30] and ACQUIRE project implemented by Engender Health in Nepal. Some of the contents in the training manual were also adapted from Maternal Infant Young Child Nutrition (MIYCN) manual prepared by Ministry of Health, Government of Nepal, Problem Management Plus (PM+) by WHO, “*Badhai*” booklet prepared by Nepal Health Education Information Communication Center; Ministry of Health, USAID and Suaahara [31].
