# Supplementary material for: The development and feasibility of a group-based household-level intervention to improve preconception nutrition in Nawalparasi district of Nepal
Source: BMC Public Health. 2022 Apr 6;22:666. doi: 10.1186/s12889-022-12980-w (PMC8984665; doi:10.1186/s12889-022-12980-w)
Supplement: Supplementary file 2 — Additional file 2. Post-intervention interview with participants:semistructured qualitative interview guide. [file 12889_2022_12980_MOESM2_ESM.docx]

**Post-intervention interview with Participants:**

**Semi structured qualitative interview guide:**

1. Can you tell me about the group intervention that you recently participated in?
   1. What did you think about it?
   2. Can you tell me some things you enjoyed about it?
   3. Can you tell me some things you did not enjoy about it?
   4. What topics did you find most useful, why?
   5. What topics did you find least useful, why?
   6. Was there anything new that you learned?
   7. What topics had you learned about before, and from who?
   8. What other topics would you liked to have learned about?
   9. Do you think that any topics were new to other people in your household, which topics and which people?
   10. Should other people in the household be involved in these groups?
2. Did you face any challenges in general with attending the groups?
   1. What were they?
      1. (probe: time, distance, resistance from family/community)
3. How did you feel about participating in groups with your husband/wife/son/daughter-in-law/mother-in-law (ask about each person individually for the appropriate person).
   1. What were some benefits? Why? Can you tell me more?
   2. What were some challenges? Why? Can you tell me more?
   3. Do you feel that it changed your relationship with anyone in the household, why and in what way?
   4. Did you talk to anyone else in your household about any of the topics that you learned? Who? Which topics?
4. If we were do to this intervention for a longer time, would you want to continue participating?
5. If we were to start this intervention again in different villages, would you recommend that we change it? How do you recommend that we change it?
   1. What other topics should we talk about?
   2. What topics should we not talk about?
   3. Was the frequency of sessions right, if not, how often should they be?
   4. Was the length of the sessions right, if not, how long should they be?
6. What did you think about the person who led the sessions, was it the right person? Is there someone else that would have been better?
   1. What about if they were led by a female community health volunteer? Would you have liked that? not liked that?
7. For women: would you have liked sessions ONLY with other newly married women?
   1. Did you like have sessions with both your husband and MIL? Would you have preferred only with your husband? Only with your MIL? Were there any topics that you wished we have talked about with certain other people? Not with certain other people?
8. For husbands: would you have liked sessions ONLY with other newly married husbands?
   1. Did you like have sessions with both your wife and mother? Would you have preferred only with your wife? Only with your mother? Were there any topics that you wished we have talked about with certain other people? Not with certain other people?
9. For MILs: would you have liked sessions ONLY with other mother-in-laws?
   1. Did you like have sessions with both your son and daughter in law? Would you have preferred only with your daughter in law? Only with your son? Were there any topics that you wished we have talked about with certain other people? Not with certain other people?
10. Is there anything else you can tell us about how to improve this?
